# Clinical characteristics and risk factors of *Helicobacter pylori* infection‐associated Sjogren's syndrome

**DOI:** 10.1002/iid3.994

**Published:** 2023-10-30

**Authors:** Ye He, Lingzhen Hu, Wei Qiu, Lixia Zhu, Xiaochun Zhu, Mingzhi Hong

**Affiliations:** ^1^ Department of Rheumatology Taizhou Municipal Hospital Taizhou Zhejiang People's Republic of China; ^2^ Department of Rheumatology The First Affiliated Hospital of Wenzhou Medical University Wenzhou Zhejiang People's Republic of China; ^3^ Department of Dermatological The First Affiliated Hospital of Wenzhou Medical University Wenzhou People's Republic of China; ^4^ Department of Burn and Plastic Surgery Taizhou Municipal Hospital Taizhou Zhejiang People's Republic of China

**Keywords:** clinical characteristics, *H. pylori* infection, multivariate analyses, risk factors, Sjögren's syndrome

## Abstract

**Objective:**

Although infectious pathogens are predominant factors for inducing and maintaining immune system disorders, there exist few reports establishing the significant correlation between *Helicobacter pylori* (*H. pylori*) infection and Sjogren's syndrome. This study aims to demonstrate the correlation between Sjogren's syndrome and *H. pylori* infection in patients, highlighting various clinical characteristics and risk factors.

**Methods:**

A single‐center retrospective observational study was conducted in patients (*n* = 224) admitted from January 1, 2012, to February 10, 2021, in the First Affiliated Hospital of Wenzhou Medical University (Wenzhou, China). All the recruited subjects with Sjogren's syndrome and *H. pylori* infection were only included by validating the available medical records online.

**Results:**

In this study, a total of 224 patients from January 1, 2012, to February 10, 2021, were diagnosed with Sjogren's syndrome. Among them, 94 patients (41.96%) with Sjogren's syndrome were infected with *H. pylori*. Accordingly, the clinical manifestations, serological and immunological characteristics, as well as gastroscopic biopsy outcomes of the recruited patients with primary Sjogren's syndrome (pSS) were reported. The multivariable analysis of the dry syndrome patients infected with *H. pylori* displayed hypergammaglobulinemia (odds ratio [OR], 0.354; 95% confidence interval [CI], 0.189‐0.663), total cholesterol (OR, 1.158; 95% CI, 0.856‐1.550), hypertension (OR, 0.227; 95% CI, 0.114‐0.455), Female sex (OR, 5.778; 95% CI, 1.458‐22.9), anti‐SSA/Ro60 positive (OR, 2.384; 95% CI, 233‐4.645), γ‐GT (OR, 0.99; 95% CI, 0.99‐1.00) and alkaline phosphatase (ALP, OR, 1.00; 95% CI, 0.99–1.00) levels.

**Conclusion:**

Together, our findings demonstrated that hypergammaglobulinemia could be the independent risk factors of *H. pylori* infection in patients with Sjogren's syndrome, requiring the physician's advice in the future.

## Graphical abstract

Histological evaluation of gastroscopy, pathology, and salivary gland biopsy specimens stained with H&E in patients with primary Sjogren's syndrome (pSS).

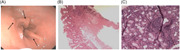

## 1 INTRODUCTION

Primary Sjogren's syndrome (pSS) refers to a chronic inflammatory autoimmune disorder, characterized by the significant infiltration of lymphocytes and destruction of exocrine glands. More often, 90% of patients with pSS suffer from the destruction of the lacrimal and salivary glands.^1^ In addition to dry eyes and xerostomia (dry mouth), pSS results in a wide range of extraglandular clinical manifestations, including abnormal dehydration, itchy skin, joint pains, diffuse parenchymal lung disease, chronic atrophic gastritis, interstitial nephritis, vasculitis, immune system‐based abnormalities, and neuropathy, among others.^2^ In this vein, several pathogens (such as coxsackie B4 virus, encephalomyocarditis virus, and *Campylobacter jejuni*) are often considered to be predominant environmental factors, leading to the substantial development of various autoimmune diseases in susceptible individuals.^3^

*Helicobacter pylori* (*H. pylori*), a Gram‐negative bacterium, infects human gastric mucosa and causes redness and inflammation, damaging the tissues in the stomach and duodenum. This most typical bacterial infection in the gastric mucosa is often related to the development of various dreadful diseases in the gastrointestinal tract (GIT), such as peptic ulcer disease, gastric cancer, and mucosa‐associated lymphoid tissue (MALT) lymphoma.^4^ Notably, *H. pylori* infection can be occurred by ingesting the organism through food or other sources, interacting with the saliva during oral transmission.^5^ In general, *H. pylori* infection in the GIT can be confirmed by various pathological tests, such as urea breath test, serological detection of anti‐*H. pylori* antibody, fecal antigen test, and urease detection of endoscopic biopsy specimens, among others.^6^

Owing to these considerations, several efforts have been dedicated to exploring the association between Sjogren's syndrome and *H. pylori* infection. The correlation between Sjogren's syndrome and *H. pylori* originated in the 1990s.^4^ Several reports further demonstrated their significant association, which, however, showed some limitations, discussed as follows.^7^, ^8^, ^9^ Despite the success in exploring the association between Sjogren's syndrome and *H. pylori* infection in the past few years, the relationship between the degree of *H. pylori* infection and the risk of Sjogren's syndrome has been very controversial. Although the prevalence of *H. pylori* infection is relatively high, the symptoms during the infection are insignificant. The long‐term existence of *H. pylori* infection substantially activates the immune system, resulting in the abridged defense against the infection. Previous reports have indicated that certain risk factors, including age, disease duration, overall disease severity, and levels of C‐reactive protein (CRP), may be significantly associated with *H. pylori* infection in patients with pSS.^10^ Of nine studies in a recent meta‐analysis, five reported the positive association of *H. pylori* infection rate with SS occurance, while four did not.^7^ However, the precise clinical characteristics and risk factors pertaining to the correlation between *H. pylori* and Sjogren's syndrome have largely remained elusive. Furthermore, the existing literature primarily focuses on exploring these risk factors among African Americans and Caucasians. To enhance our understanding of the clinical characteristics and risk factors in Chinese patients with Sjogren's syndrome infected by *H. pylori*, we conducted a single‐center retrospective observational study at the First Affiliated Hospital of Wenzhou Medical University in Wenzhou, China. The study included a total of 224 patients who were admitted between January 1, 2012, and February 10, 2021. We examined the patients' clinical manifestations, immunological and serological profiles, as well as the outcomes of gastroscopic biopsies in individuals with pSS. Our findings may lead to a better understanding of SS patients who also have gastrointestinal issues and offer suggestions for specialised care.

## 2 METHODS

### 2.1 Study design and subjects

In this study, we demonstrated a retrospective cross‐sectional study based on the registered patients with pSS between January 1, 2012, to February 10, 2021, in the First Affiliated Hospital of Wenzhou Medical University. Notably, this Hospital of Wenzhou Medical University is a 3380‐bed tertiary hospital, serving as one of the largest healthcare centers in Zhejiang, PR China. Before conducting the experiment, human research ethics approval was obtained (No. 2021‐R051) from the Ethical Committee of the First Affiliated Hospital of Wenzhou Medical University. Notably, the organizers have ensured that the personal data would be kept confidential. Moreover, the Ethics Committee eventually determined that patient consent was not required as it was a retrospective study. In addition, patients were not required to provide any license statement due to being devoid of personal information.

**Figure 1:**
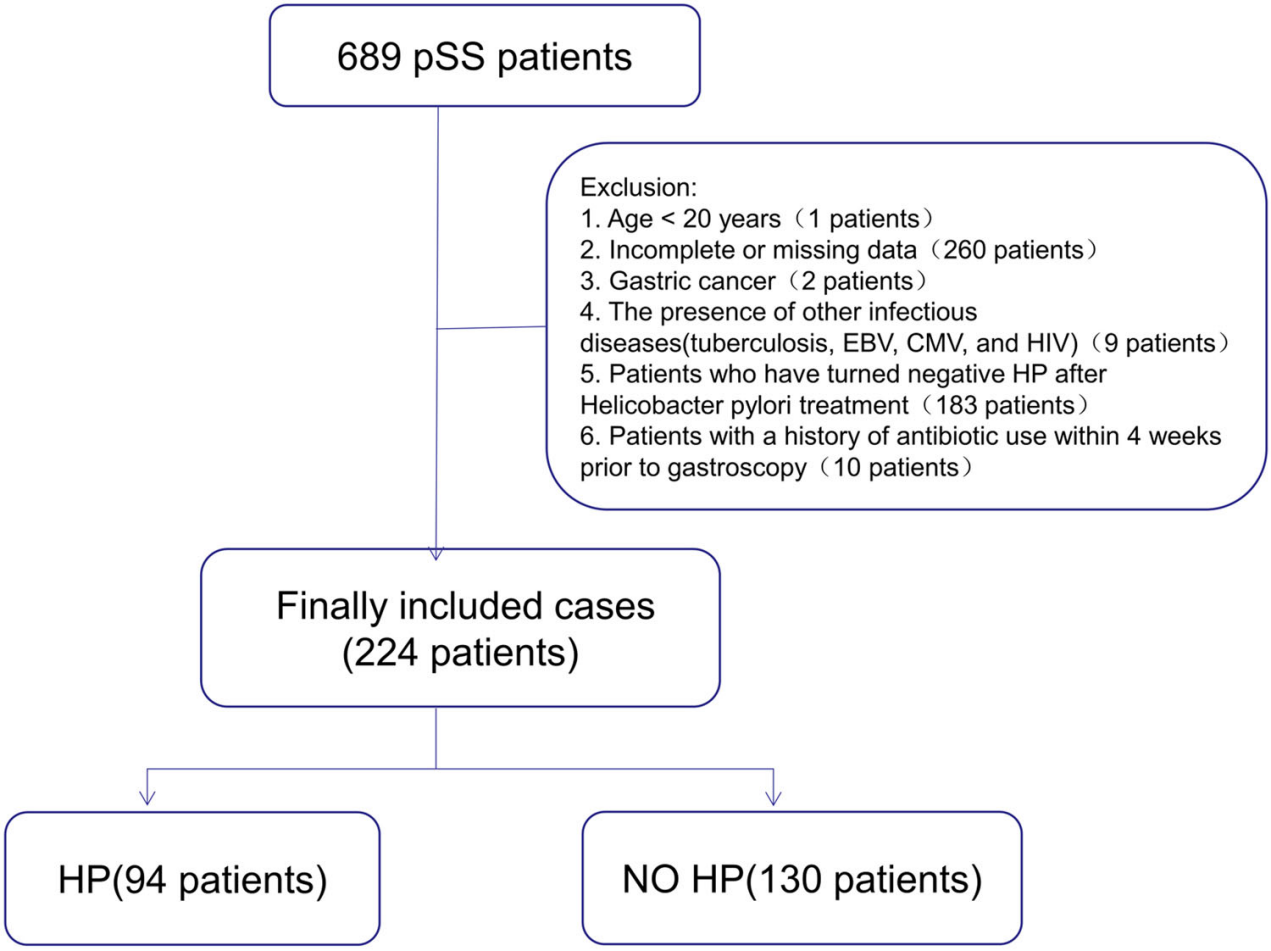
Flow diagram of patient selection.

In the case of a patient with pSS advised of gastroscopy more than once, the gastroscopy results closest to the time of diagnosis of pSS were retained. Notably, the exclusion criteria were set as follows: (a) The age of patients lesser than 20 years old; (b) patients with incomplete or missing data; (c) patients diagnosed with gastric cancer; (d) patients diagnosed with other infectious diseases (tuberculosis, Epstein‐Barr virus, EBV, cytomegalovirus, CMV, and human immunodeficiency virus, HIV); (e) patients with negative *H. pylori* infection result after systemic *H. pylori* treatment; and (f) Patients with a history of antibiotic usage within 4 weeks before gastroscopy. Considering these criteria, a total of 689 patients were first included in this study, along with the subjects who satisfied the 2002 classification criteria of the American‐European Consensus Group (AECG) for pSS.^11^ Further, the final count of 224 cases was recruited, of which 94 cases were *H. pylori*‐positive pSS and 130 cases were *H. pylori*‐negative pSS patients (Figure 1).

### 2.2 Data collection

As mentioned earlier, the patients' records were collected by reviewing and validating the electronic medical records available in the database. Accordingly, the collected medical records were sorted out concerning the demographic data (age and gender) and the clinical data, including underlying diseases, xerophthalmia, xerostomia, articular, cutaneous, pulmonary, renal, gastrointestinal, and endocrine involvements. Further, the immunological tests were performed by using various commercially available kits based on the immunoblotting approaches, such as antinuclear antibodies (ANA), anti‐Ro antibodies, anti‐SSA antibodies, anti‐La/SSB antibodies, as well as enzyme‐linked immunosorbent assay (ELISA) techniques, including rheumatoid factor (RF), complement component 3 (C3) or C4, immunoglobulin (Ig) G, IgA, and IgM levels. It should be noted that patients who received the C13/C14 puff tests and confirmed as *H. pylori* positive in official test reports in other hospitals were also categorized as the *H. pylori* infection group.

### 2.3 Definitions

According to the records, the cases were diagnosed based on the 2002 classification criteria of the AECG for pSS.^12^ Therefore, the confirmed pSS cases of *H. pylori* infection were determined based on the positive result of a 13 C *urea breath test* or a combined antral and corpus *rapid urease* testing protocol of gastroscopy.^13^

### 2.4 Statistical analysis

The statistical analysis was performed using the SPSS 19.0 software (IBM Corp.). The data of continuous variables were presented as mean ± standard deviation (SD) in the case of normal distribution. Contrarily, the data were presented as the median and interquartile range (IQR) in the case of nonnormal distribution. Accordingly, the continuous variables were analyzed by the Student's *t*‐test or Mann–Whitney *U* test. To this end, the data of enumeration variables were compared by Pearson's *χ* ^2^ or Fisher exact tests. The variables with a defined significance level of *p* < 0.05 in the univariate analysis were considered appropriate candidates for building step‐wise logistic regression multivariable models. Notably, a two‐tailed test at a *p* < 0.05 level was considered statistically significant.

## 3 RESULTS

### 3.1 Demographic and clinical characteristics

The demographic features (age and gender) and clinical characteristics, including underlying diseases of the selected subjects, are summarized in Table 1. Among the recruited 224 pSS patients, 94 were diagnosed with *H. pylori* infection, accounting for 41.96%, and the rest of the pSS patients (130, 58.04%) showed no *H. pylori* infection. The statistics showed that the mean age of *H. pylori*‐positive subgroup (47.1 ± 11.1) was significantly lower than *H. pylori*‐negative subgroup (55 ± 12.1). Moreover, the mean disease duration of *H. pylori*‐positive and *H. pylori*‐negative was over 11 and 12 months in the cohort, respectively, with no significant difference between the two subgroups. In virtue of the clinical characteristics, except hypertension, two subgroups (*H. pylori*‐positive and *H. pylori*‐negative) displayed similar proportions of diabetes mellitus, interstitial lung disease, and cardiac involvement (*p* > 0.05). In addition, the general symptoms, such as xerophthalmia, xerostomia, fever, fatigue, and arthralgia in both subgroups, showed no substantial discrepancy. Moreover, no significant difference in total ESSDAI scores between the two groups was observed (Table 1).

**Table 1:** Baseline characteristics of patients with *Helicobacter pylori*‐positive pSS and *H. pylori*‐negative pSS patients.

Characteristics	Total (*n* = 224)	*H. pylori*‐positive pSS patients (*n* = 94)	*H. pylori*‐negative pSS patients (*n* = 130)	*p* Value
Female sex, *n* (%)	202 (90.2)	91 (96.8)	111 (85.4)	**0.005**
Age, y (mean + SD)	51.7 ± 12.3	47.1 ± 11.1	55 ± 12.1	**0.000**
Hypertension, *n* (%)	69 (30.8)	42 (44.7)	27 (20.8)	**0.000**
Disease duration, (months) (IQR)	12 (4–34.5)	11 (3–30.8)	12 (5–36)	0.273
Diabetes mellitus, *n* (%)	11 (4.9)	2 (2.1)	9 (6.9)	0.185
Xerostomia, *n* (%)	107 (47.8)	47 (50)	60 (46.2)	0.570
Xerophthalmia, *n* (%)	114 (50.9)	53 (56.4)	61 (46.9)	0.162
Fever, *n* (%)	22 (9.8)	8 (8.5)	14 (10.8)	0.575
Fatigue, *n* (%)	39 (17.4)	15 (16.0)	24 (18.5)	0.626
Arthralgia, *n* (%)	72 (32.1)	26 (27.7)	46 (35.4)	0.222
ESSDAI (IQR)	7.5 (3–16)	6 (3–14)	8.5 (3–19)	0.098
Glandular domain involvement, *n* (%)	7 (3.1)	3 (3.2)	4 (3.1)	1.000
Cardiac involvement, *n* (%)	64 (28.6)	26 (27.7)	38 (29.2)	0.797
LSGB, Lymphocytic focus ≥1, *n* (%)	142 (63.4)	65 (69.1)	77 (59.2)	0.128
Interstitial lung diseases, *n* (%)	53 (23.7)	19 (20.2)	34 (26.2)	0.302

*Note*: Bold indicates *p* < 0.05.

Abbreviations: disease duration, disease duration measured from the day diagnosed; ESSDAI, European League Against Rheumatism Sjögren's syndrome disease activity index; IQR, interquartile range; LSGB, labial salivary gland biopsy.

### 3.2 Biological indicators

Further, a comparison of various biological indicators based on the complete blood picture, enumerating various components of blood, between the groups of *H. pylori*‐positive pSS and *H. pylori*‐negative pSS, is presented in Table 2. Compared with *H. pylori*‐negative pSS group, patients with *H. pylori*‐positive pSS group displayed a lower hematocrit (median g/L, 42.95 vs. 41.19, *p* = .013), glutamic‐pyruvic transaminase (GPT) (median U/L, 16 vs. 8, *p* = .017), ALP (median U/L, 64 vs. 77, *p* = .002) and γ‐glutamyl transpeptidase (γ‐GT, median U/L, 18 vs. 25.5, *p* = .001). In terms of lipid levels in serum, the total cholesterol content of *H. pylori*‐positive patients was significantly higher than that of *H. pylori*‐negative patients: 5.31 (SD = 1.15) versus 4.83 (SD = 1.37), high‐density lipids (HDL): 1.30 (SD = 0.37) versus 1.15 (SD = 0.39), low‐density lipids (LDL, median mmol/L, 3.39 vs. 3, *p* = .022). Nonetheless, no significant difference between the two groups was observed concerning the albumin, GPT, glutamic‐oxaloacetic transaminase (GOT), blood routine test, lactate dehydrogenase (LDH), serum creatinine, uric acid, triglycerides, HDL, and creatine kinase levels. Moreover, no significant difference was observed between the two subgroups in terms of anti‐SSA/Ro52‐positive, anti‐La/SSB‐positive, RF positivity, and complement 3, as well as complement 4 prevalence. Contrarily, the incidence rates of anti‐SSA/Ro60‐positive and hypergammaglobulinemia were much higher, while CRP was slightly higher in *H. pylori*‐positive infection compared to *H. pylori*‐negative pSS patients.

**Table 2:** Comparison of biological indicators between groups of *Helicobacter pylori*‐positive pSS and *H. pylori*‐negative pSS.

Biological indicators	Total (*n* = 224)	*H. pylori*‐positive pSS patients (*n* = 94)	*H. pylori‐*negative pSS patients (*n* = 130)	*p* Value
Blood routine test				
WBC (×10^9^/L) (IQR)	5.28 (4.28–6.33)	5.38 (4.32–6.26)	5.21 (4.27–6.34)	0.586
Neutrophil numerals (×10^9^/L) (IQR)	2.89 (2.17–4.18)	2.96 (2.20–4.32)	2.77 (2.14–4.07)	0.599
Lymphocytes (×10^9^/L) (IQR)	1.59 (1.21–2.01)	1.58 (1.26–2.15)	1.59 (1.17–1.92)	0.168
Hemoglobin (g/L) (IQR)	123.65 (118.00–134.75)	125.00 (119.75–135.00)	126.00 (117.75–134.25)	0.975
Platelet (×10^9^/L) (IQR)	206.50 (172.00–246.75)	203.50 (175.25–247.50)	207.09 (169.00–246.25)	0.891
**Liver and kidney function**				
Albumin (g/L) (IQR)	41.30 (37.10–44.20)	40.8 (37.1–43.6)	42.10 (36.80–44.40)	0.23
GPT (U/L) (IQR)	17.00 (13.00–27.00)	16.00 (12.00–25.00)	16.00 (13.00–28.00)	0.17
GOT (U/L) (IQR)	23.00 (19.00–30.00)	22.00 (19.00–28.00)	23.50 (19.00–32.25)	0.075
ALP (U/L) (IQR)	73.00 (58.25–85.75)	64.00 (54.00–83.33)	77.00 (62.00–93.00)	**0.002**
γ‐GT (U/L) (IQR)	21.00 (14.25–43.57)	18.00 (14.00–27.25)	25.50 (15.00–48.50)	**0.001**
LDH (U/L) (IQR)	217.39 (195.25–217.39)	217.39 (216.54–217.39)	217.39 (190.50–217.39)	0.324
SCr (μmol/L) (IQR)	58.00 (51.00–65.00)	56.00 (50.00–64.25)	58 (51.00–65.25)	0.365
Uric acid (μmol/L) (IQR)	281.00 (231.00–329.00)	286.00 (234.00–315.00)	274 (223–345)	0.923
Total cholesterol (mmol/L) (mean ± SD)	5.02 ± 1.30	5.31 ± 1.15	4.83 ± 1.37	**0.015**
Triglycerides (mmol/L) (IQR)	1.43 (0.99–1.56)	1.46 (1.01–1.48)	1.42 (0.98–1.59)	0.946
HDL (mmol/L) (IQR)	1.2 (1.02–1.39)	1.2 (1.13–1.49)	1.23 (0.97–1.31)	0.175
LDL (mmol/L) (IQR)	3.13 (2.22–3.79)	3.39 (2.48–3.79)	3.00 (2.11–3.79)	**0.022**
Creatine kinase (U/L) (IQR)	97.00 (52.25–108.65)	108.32 (62.25–108.65)	88.00 (45.00–108.65)	0.286
ANA positive, *n* (%)	214 (95.5)	90 (95.7)	61 (63.85)	**0.001**
*n* (%)	143 (63.8)	68 (72.3)	75 (57.7)	**0.024**
Anti‐SSB positive, *n* (%)	63 (28.1)	29 (30.9)	34 (26.2)	0.440
Anti‐Ro52 positive, *n* (%)	149 (66.5)	65 (69.1)	84 (64.6)	0.478
Hypergammaglobulinemia (>16 g/L) *n* (%)	116 (51.8)	59 (62.8)	57 (43.8)	**0.005**
IgA (g/L)(IQR)	2.99 (2.24–3.86)	3.00 (2.27–4.02)	2.98 (2.24–3.82)	0.909
IgM (g/L) (IQR)	1.25 (0.90–1.70)	1.27 (0.90–1.71)	1.25 (0.91–1.74)	0.675
C3 level (g/L) (IQR)	1.06 (0.92–1.16)	1.05 (0.91–1.16)	1.07 (0.93–1.17)	0.740
C4 level (g/L) (IQR)	0.22 (0.17–0.24)	0.21 (0.17–0.23)	0.23 (0.18–0.26)	0.133
RF (IU/mL) (IQR)	14.65 (9.5–60.92)	20.00 (9.50–60.92)	13.45 (9.69–60.92)	0.816
ESR (mm/h) (IQR)	20.50 (10.00–28.00)	20.5 (11.00–28.25)	20.00 (9.00–26.25)	0.510
CRP (mg/L) (IQR)	3.13 (2.74–5.65)	3.13 (3.02–6.51)	3.02 (1.97–5.03)	**0.041**
Dimer (mg/L）(IQR)	1.36 (0.35–1.45)	1.29 (0.35–1.45)	1.45 (0.36–1.46)	0.634
Fasting blood glucose (mmol/L) (IQR)	5.50 (4.93–5.59)	5.30 (4.90–5.56)	5.56 (5.0–5.60)	0.343

*Note*: Bold indicates *p* < 0.05.

Abbreviations: ALP, alkaline phosphatase; ANA, antinuclear antibodies; ANC, absolute neutrophil count; C3, complement component 3; C4, complement; CRP, C‐reactive protein; ESR, erythrocyte sedimentation rate; GOT, glutamic‐oxaloacetic transaminase; GPT, glutamic‐pyruvic transaminase; IQR, interquartile range; LDH, lactic dehydrogenase; RF, rheumatoid factor; SCr, serum creatinine; WBC, white blood count; γ‐GT, gamma‐glutamyl transpeptidase.

### 3.3 Characteristics of high titer ANA‐positive patients in *H. pylori*‐positive pSS

As depicted in Table 3, it was observed that the high titers of ANA levels (1:320) were detected in 49 *H. pylori*‐positive pSS patients, accounting for 52.13%. As anticipated, the ANA‐high subgroup showed positive histological findings of labial salivary gland biopsy (LSGB). Compared to the ANA‐low subgroup, the ANA‐high subgroup showed higher anti‐SSA60/SSB and RF positive rates and augmented IgG levels. In addition, the ANA‐high subgroup manifested more severe renal impairment with significantly higher and uric acid levels but no significant difference in ESSDAI concerning the ANA‐low subgroup. However, such characteristics of high titer were not found in the patients in *H. pylori*‐negative pSS (Supporting Information: Table S1).

**Table 3:** Analysis of multiple features of *Helicobacter* *pylori*‐positive pSS according to ANA positivity.

Characteristics	Total (*n* = 94)	ANA‐low (*n* = 45)	ANA‐high (*n* = 49)	*p* Value
Female sex, *n* (%)	91 (96.8)	44 (97.8)	47 (95.9)	1.00
Age, median years (mean ± SD)	47.1 ± 11.1	48.7 ± 8.4	45.7 ± 13.0	0.18
Hypertension, *n* (%)	42 (44.7)	19 (42.2)	23 (46.9)	0.65
ESSDAI	6 (3.14)	6 (212.5)	6 (4.15)	0.30
Disease duration (months) (IQR)	11 (3–31)	10 (3.5–23)	12 (1.5–37)	0.85
LSGB, lymphocytic focus ≥ 1, *n* (%)	65 (69.1)	18 (40.0)	47 (95.9)	**0.00**
Anti‐SSA/Ro60 positive, *n* (%)	68 (72.3)	28 (62.2)	40 (81.6)	**0.036**
Anti‐Ro52 positive, *n* (%)	65 (69.1)	27 (60.0)	38 (77.6)	0.07
Anti‐SSB positive,*n* (%)	29 (30.9)	9 (20.0)	20 (40.8)	**0.029**
IgG levels, (g/L) (IQR)	16.9 (14.5–19.7)	16.4 (13.95–17.8)	17.5 (15.1–20.5)	**0.02**
Hypergammaglobulinemia (>16 g/L), *n* (%)	59 (62.8)	25 (55.6)	34 (69.4)	0.17
IgA levels (g/L) (mean ± SD)	3.07 ± 1.13	2.99 ± 1.17	3.16 ± 1.10	0.47
IgM levels (g/L) (IQR)	1.27 (0.90–1.71)	1.29 (0.96–1.73)	1.25 (0.83–1.7)	0.67
C3 level (g/L) (IQR)	1.05 (0.91–1.16)	1.03 (0.92–1.12)	1.07 (0.90–1.17)	0.60
C4 level (g/L) (IQR)	0.21 (0.17–0.23)	0.22 (0.18–0.25)	0.19 (0.17–0.23)	0.06
RF positive, *n* (%)＞15.9 IU/mL	48 (51.1)	18 (40.0)	30 (61.2)	**0.04**
WBC (×10^9^/L) (IQR)	5.38 (4.32–6.26)	5.34 (4.30–6.82)	5.43 (4.33–6.12)	0.84
Neutrophil numerals (×10^9^/L) (IQR)	2.96 (2.20–4.32)	2.44 (2.12–4.25)	2.98 (2.45–4.71)	0.28
*Lymphocytes* (×10^9^/L) (IQR)	1.58 (1.26–2.15)	1.70 (1.38–2.21)	1.56 (1.19–2.04)	0.33
Hemoglobin (g/L) (IQR)	125 (119.75–135)	128 (118–136)	123 (120–131)	0.30
Platelet (×10^9^/L) (mean ± SD)	207.99 ± 69.65	211.44 ± 61.77	204.82 ± 76.68	0.65
Albumin (g/L) (IQR)	40.8 (37.1–43.6)	40.8 (35.8–44.1)	40.7 (38.6–43.2)	0.73
GPT (U/L) (IQR)	16 (12.75–25)	16 (12.5–25)	15 (12.5–24.5)	0.95
GOT (U/L) (IQR)	22 (19–28)	22 (17.5–29)	23 (20–27)	0.37
γ‐GT (U/L) (IQR)	18 (14–27.25)	17 (14–27)	18 (14–27.5)	0.74
ALP (U/L) (IQR)	64 (54–83.3)	67 (53–86)	63 (54–79)	0.67
SCr (μmol/L) (IQR)	56 (50–64.25)	53 (49.5–62.8)	61 (50.5–69)	**0.04**
LDH (U/L) (IQR)	217.39 (216.54–217.39)	217.39 (206–217.39)	217.39 (217.39–217.39)	0.50
CRP (mg/L) (IQR)	3.02 (1.43–3.3)	3.02 (1.03–4.15)	3.02 (3.02–3.30)	0.30
ESR (mm/h) (IQR)	20 (11–28.3)	20 (9–24.5)	21 (13–31.5)	0.33
D‐dimer (mg/L) (IQR)	1.2 (0.33–1.45)	1.12 (0.34–1.45)	1.28 (0.3–1.45)	0.92
Fasting blood glucose (mmol/L)(IQR)	5.35 (4.8–5.56)	5.2 (4.6–5.56)	5.56 (5–5.56)	0.07
Uric acid (μmol/L) (IQR)	285.5 (233.5–315)	252 (224.5–304)	308.7 (264–351)	**0.00**
Total cholesterol (mmol/L) (IQR)	5.05 (4.67–5.93)	5.05 (4.6–6.09)	5.05 (4.65–5.65)	0.54
Triglycerides (mmol/L) (IQR)	1.46 (1.01–1.48)	1.43 (0.94–1.57)	1.48 (1.07–1.48)	0.80
HDL (mmol/L) (IQR)	1.20 (1.13–1.49)	1.27 (1.02–1.6)	1.2 (1.16–1.36)	0.42
LDL (mmol/L) (IQR)	3.39 (2.48–3.79)	3.34 (2.19–3.79)	3.43 (2.55–3.79)	0.85
Creatine kinase (U/L) (IQR)	108.65 (62.25–108.65)	93 (56.5–108.65)	108.65 (63.5–108.65)	0.59

*Note*: Bold indicates *p* < 0.05.

Abbreviations: ANA, antinuclear antibodies; C3, complement 3; C4, complement 4; CCR, creatinine clearance rate; CRP, C‐reactive protein; ESR, erythrocyte sedimentation rate; ESSDAI, European League Against Rheumatism Sjögren's syndrome disease activity index; LSGB, labial salivary gland biopsy; RF, rheumatoid factor; SCr, serum creatinine.

### 3.4 Gastroscopic findings in *H. pylori*‐positive pSS patients

**Figure 2:**
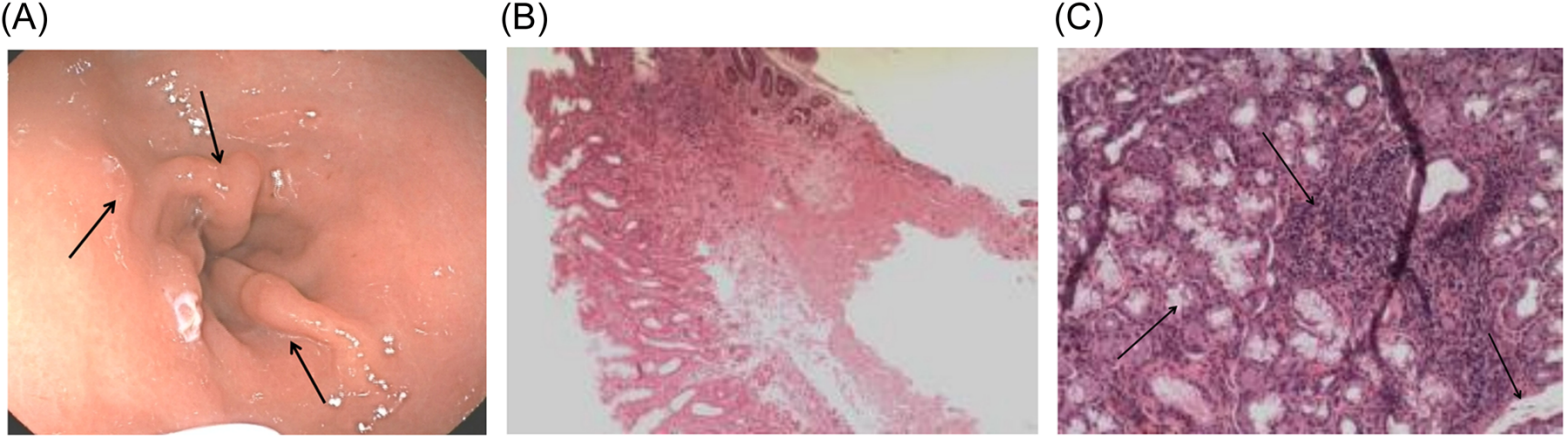
Histological evaluation of gastroscopy, pathology, and salivary gland biopsy specimens stained with hematoxylin and eosin in patients with primary Sjogren's syndrome (pSS). (A) Gastroscopy shows hyperplastic polyps in pSS patients infected with Helicobacter pylori (arrow) (B) Gastroscopic pathology of pSS patients with Helicobacter pylori infection showed intestinal metaplasia and atrophy (C) Lip gland biopsy specimens from pSS patients infected with Helicobacter pylori showing dilation of ducts in lobules, atrophy of a large number of acini, and lymphocytic infiltration in stroma (arrow; H&E, 200 X).

Among the recruited subjects, 162 pSS patients with *H. pylori*‐positive underwent gastroscopy and pathological examinations. Among them, 58 pSS patients were diagnosed with *H. pylori* infection. Generally, atrophy without metaplasia is the predominant clinical manifestation of gastroscopy in *H. pylori*‐negative pSS patients. At the same time, the most common pathological manifestations in *H. pylori*‐positive pSS patients include intestinal metaplasia and atrophy with metaplasia. As shown in Table 4 and Figure 2, the respective gastroscopic manifestations in both groups were presented.

**Table 4:** Gastroscopic manifestations of pSS with *Helicobacter pylori*‐positive.

Characteristics	Total (*n* = 162)	*H. pylori*‐negative pSS patients (*n* = 104)	*H. pylori*‐positive pSS patients (*n* = 58)	*p* Value
Foveolar‐hyperplastic polyp *n* (%)	22 (13.6)	19 (18.3)	3 (5.2)	**0.036**
Xanthoma, *n* (%)	3 (1.9)	2 (1.9)	1 (1.7)	1.00
Intestinal metaplasia, *n* (%)	60 (37.0)	26 (25.0)	34 (58.6)	**0.00**
Depressive erosion, *n* (%)	110 (67.9)	66 (63.5)	44 (75.9)	0.11
Fundic gland poly, *n* (%)	3 (1.9)	3 (2.9)	0 (0)	0.49
Atrophy without metaplasia, *n* (%)	136 (84.0)	101 (97.1)	35 (60.3)	**0.00**
Atrophy with metaplasia, *n* (%)	26 (16.0)	3 (2.9)	23 (39.7)	**0.00**

*Note*: Bold indicates *p* < 0.05.

Abbreviation: pSS, primary Sjogren's syndrome.

### 3.5 Different histological patterns of LSGB in *H. pylori*‐positive pSS patients

As depicted in Table 5, the positive histological findings with the lymphocytic focus score (FS) of ≥1 were observed in 69.1% of the *H. pylori*‐positive pSS patients. Moreover, the histologically positive group showed more frequent ANA ≥ 1:320, anti‐SSA/Ro60 positive, anti‐Ro52 positive, and anti‐SSB positive prevalence, compared to its contrary group. Interestingly, the positive group resulted in much higher levels of uric acid (*p* < 0.05). Together, these indices in the patients of the positive subgroup perhaps suggested more severe conditions than the negative subgroup. However, no different histological patterns of LSGB were found in *H. pylori*‐negative pSS patients (Supporting Information: Table S2).

**Table 5:** Analysis of multiple features of *Helicobacter pylori*‐positive pSS according to histological pattern of LSGB.

Characteristics	Total (*n* = 94)	LSGB negative (*n* = 29)	LSGB positive (*n* = 65)	*p* Value
Female sex, *n* (%)	91 (96.8)	29 (100)	62 (95.38)	0.59
Age, median years (mean ± SD)	47.12 ± 11.12	47.41 ± 10.93	46.98 ± 11.28	0.86
Disease duration (months) (IQR)	11 (3–30.75)	8 (4–15)	12 (2–36)	0.86
Fatigue, *n* (%)	15 (15.96)	6 (20.69)	9 (13.85)	0.59
ESSDAI (IQR)	6 (3–14)	9 (3.5–14.5)	6 (3–13)	0.27
ANA‐positive, ANA ≥ 1:320, *n* (%)	49 (52.13)	9 (31.03)	40 (61.54)	**0.006**
Anti‐SSA/Ro60 positive, *n* (%)	68 (72.3)	16 (55.17)	52 (80)	0.013
Anti‐Ro52 positive, *n* (%)	65 (69.15)	14 (48.28)	51 (78.46)	**0.003**
Anti‐SSB positive, *n* (%)	29 (30.85)	3 (10.34)	26 (40)	**0.004**
Hypergammaglobulinemia (>16 g/L), *n* (%)	58 (61.7)	17 (58.6)	41 (63.1)	0.68
IgA levels (g/L) (mean ± SD)	3.08 ± 1.13	2.95 ± 1.02	3.13 ± 1.18	0.48
IgM levels, g/L(IQR)	1.27 (0.90–1.71)	1.3 (1.05–1.68)	1.19 (0.86–1.79)	0.44
C3 level (g/L) (IQR)	1.05 (0.91–1.16)	1.07 (0.95–1.19)	1.04 (0.88–1.16)	0.47
C4 level (g/L) (IQR)	0.21 (0.17–0.23)	0.22 (0.18–0.23)	0.21 (0.17–0.24)	0.39
RF (IU/mL) (IQR)	20 (9.5–60.92)	11 (8.88–60.92)	20 (9.5–60.92)	0.17
WBC (×109/L) (IQR)	5.38 (4.32–6.26)	5.46 (4.46–7.04)	5.34 (4.17–6.19)	0.38
Hemoglobin (g/L) (IQR)	125 (119.8–135)	127 (119.5–136.0)	124 (119.5–134.5)	0.58
Platelet (×109/L) (IQR)	203.5 (175.3–247.5)	218 (174.5–275.5)	200 (175.5–240.5)	0.22
GPT (U/L) (IQR)	16 (12.8–25)	18 (14–24.5)	15 (12–25)	0.30
GOT (U/L) (IQR)	22 (19–28)	24 (21–30.5)	22 (18.5–27)	0.09
γ‐GT (U/L) (IQR)	18 (14–27.3)	18 (14.5–27)	18 (14–27.5)	0.93
ALP (U/L) (IQR)	64 (54–79.8)	63 (56–84)	66 (54–80.5)	0.80
Uric acid (μmol/L)(IQR)	285.5 (233.5–315)	265 (212.5–307.37)	298 (248.5–334.5)	**0.031**
CRP (mg/l) (IQR)	3.02 (1.43–3.3)	3.13 (3.02–6.66)	3.02 (1.14–3.30)	0.08
SCr (μmol/L) (IQR) (IQR)	56 (50–64.3)	56 (49.5–64)	57 (50–65)	0.43
d‐dimer (mg/L) (IQR)	1.2 (0.33–1.45)	1.45 (0.58–1.45)	0.99 (0.30–1.45)	0.15
ESR (mm/h) (IQR)	20 (11–28.5)	15 (8.5–31)	21 (13–28.5)	0.45
Fasting blood glucose (mmol/L) (IQR)	5.35 (4.8–5.57)	5.4 (5.0–5.58)	5.3 (4.75–5.56)	0.28

*Note*: Bold indicates *p* < 0.05.

Abbreviations: ANA, antinuclear antibodies; C3, complement 3; C4, complement 4; CRP, C‐reactive protein; ESR, erythrocyte sedimentation rate; ESSDAI, European League Against Rheumatism Sjögren's syndrome disease activity index; ESSPRI, European League Against Rheumatism Sjögren's syndrome patient reported index; IQR, interquartile range; LSGB, labial salivary gland biopsy; pSS, primary Sjogren's syndrome; RF, rheumatoid factor; SCr, serum creatinine.

### 3.6 Independent risk factors for *H. pylori*‐positive pSS patients

Further, a univariate analysis was performed, exploring the association of a series of variables with neurological involvement. As shown in Table 6, a series of variables was associated, including hypertension, sex, anti‐SSA/Ro60 positivity, IgG levels, ALP, γ‐GT, and total cholesterol (*p* < 0.05). To avoid their interference from potentially related factors, these associated factors related to *H. pylori* infection were further analyzed through the multivariate analysis (Table 7). In this framework, the multivariate logistic regression model analysis showed that the independent risk factors of *H. pylori* infection in pSS included hypergammaglobulinemia (OR, 0.354; 95% CI, 0.189‐0.663), hypertension (OR, 0.227; 95% CI, 0.114‐0.455), female sex (OR, 5.778; 95% CI, 1.458‐22.9), anti‐SSA/Ro60 positive (OR, 2.384; 95% CI, 233‐4.645), γ‐GT (OR, 0.99; 95% CI, 0.99‐1.00).

**Table 6:** Univariate analysis of related factors of *Helicobacter pylori* infection in pSS patients.

Variable	Odds ratio	95% confidence interval	*p* Value
LSGB, lymphocytic focus ≥1, *n* (%)	0.65	0.37–1.14	0.13
Hypertension, *n* (%)	0.325	0.180–0.584	<0.01
Female sex, *n* (%)	5.192	1.489–18.001	0.01
ANA positive, ANA ≥ 1:320, *n* (%)	0.843	0.477–1.381	0.442
Anti‐SSA/Ro60 positive, *n* (%)	0.52	0.30–0.92	0.025
Anti‐SSA/Ro52 positive, *n* (%)	0.82	0.46–1.44	0.48
Anti‐SSB positive, *n* (%)	0.79	0.44–1.43	0.44
Hypergammaglobulinemia, >16 g/L, *n* (%)	0.463	0.269–0.833	0.005
ALP (U/L) (IQR)	1.00	0.98–1.00	0.018
γ‐GT (U/L) (IQR)	0.99	0.99–1.00	0.03
Total cholesterol (mmol/L)	1.30	1.02–1.65	0.03
HDL (mmol/L)	2.170	0.987–4.768	0.054
LDL (mmol/L)	0.99	0.95–1.03	0.57
CRP (mg/L, mean ± SD)	0.98	0.95–1.00	0.099

Abbreviations: ALP, alkaline phosphatase; ANA, antinuclear antibodies; CRP, C‐reactive protein; HDL, high‐density lipoprotein; LDL, low‐density lipoprotein; LSGB, labial salivary gland biopsy; pSS, primary Sjogren's syndrome; γ‐GT, γ‐glutamyl transpeptidase.

**Table 7:** Multivariable analysis of factors associated with *Helicobacter pylori* infection in pSS patients.

Variable	Odds ratio	95% confidence interval	*p* Value
Hypertension, *n* (%)	0.227	0.114‐0.455	<0.01
Female sex, *n* (%)	5.778	1.458‐22.90	0.013
Anti‐SSA/Ro60 positive, *n* (%)	2.384	1.233‐4.645	0.011
Hypergammaglobulinemia, >16 g/L, *n* (%)	0.354	0.189‐0.663	0.001
ALP (U/L) (IQR)	1.00	0.99‐1.00	0.082
γ‐GT (U/L) (IQR)	0.99	0.99‐1.00	0.04
Total cholesterol (mmol/L)	1.158	0.856‐1.550	0.323

Abbreviations: ALP, alkaline phosphatase; pSS, primary Sjogren's syndrome.

## 4 DISCUSSION

This study chiefly aims to demonstrate the condition of pSS patients in the cohort infected with *H. pylori* in China. Further, we intend to explore the association between pSS and *H. pylori* in terms of clinical characteristics and risk factors. Considering these aspects and relevance to the designed study, several significant results were discovered in the current study. Initially, it should be noted that patients with pSS are often infected with *H. pylori*, leading to some clinical characteristics and risk factors. In this study, it was observed that several risk factors were associated with the *H. pylori* infection, including age, gender, hypertension, ALP, γ‐GT, total cholesterol, LDL, anti‐SSA/Ro60 positive, hypergammaglobulinemia, and CRP levels (Tables 1 and 2). In addition, several *H. pylori*‐positive pSS patients showed obvious metaplasia with atrophy (Table 4). Finally, a multivariate logistic regression model analysis indicated that the independent risk factors of *H. pylori* infection in pSS patients included hypergammaglobulinemia, hypertension, female sex, Anti‐SSA/Ro60 positive and γ‐GT. Contrarily, the ALP levels were negatively correlated with the *H. pylori* infection in pSS patients.

Although several investigations have been reported the correlation between *H. pylori* infection and Sjogren's syndrome demonstrated a controversial conclusion. A recent meta‐analysis involving more than 600 Sjogren's syndrome patients revealed that Sjogren's syndrome patients had a slightly greater rate of *H. pylori* infection than healthy controls.^7^ Additionally, serum antibody titers to HP substantially linked with illness activity index, age, disease duration, and C‐reactive protein levels in people with Sjogren's syndrome.^8^, ^9^ However, a previous study on the seroprevalence of ARVs in wild birds reported none H. pylori seropositivity associated with primary SS in 164 Swedish patients.^5^ In the current study, we observed a high proportion (41.96%) of *H. pylori* infection in pSS patients. Similarly, a recent report on the meta‐analysis of patients with pSS demonstrated a significantly higher rate of *H. pylori* infection in pSS than healthy control samples.^7^ Nonetheless, the rigorous explanations regarding the high infection rates of *H. pylori* remained unclear. Some researchers concluded that the reasons behind the high infection rates of *H. pylori* might be associated with the persistent presence of *H. pylori*. Further, the presence of *H. pylori* could substantially activate the human immune system, producing abundant levels of cytokines, augmenting the infiltration of gastric mucosa by neutrophils and macrophages, and producing effector T cells and antibodies.^14^ In this framework, previous reports demonstrated several mechanistic views regarding the induction of autoimmune pathogenesis by *H. pylori*, including cessation of the immunological tolerance by bacteria‐derived heat shock protein 60 (HSP60), microchimerism, molecular imitation, and substantial activation of autoreactive T cells, and immunological response to *H. pylori* antigens.^9^

Similar to the reported studies,^15^, ^16^, ^17^, ^18^ various risk factors relevant to the *H. pylori* infection in patients with pSS were observed, such as female proportion, hypertension, higher total cholesterol, higher LDL, high CRP, and hypergammaglobulinemia (Tables 1 and 2). In one case, a positive correlation existed between age and *H. pylori* infection, in which older patients with pSS showed a higher prevalence of *H. pylori* infection than the average population.^8^ These findings were contrary to our findings. The differences might be related to genetic, regional reasons, or eating habits. Notably, the estimations and precise reasons behind the differences are worthy of further investigation. Compared with the pSS group without *H. pylori* infection, *H. pylori*‐infected pSS group of patients displayed lower ALP levels, lower GGT, and higher anti‐SSA/ro60 positivity (Tables 1 and 2). In previous studies, *H. pylori* infection was positively correlated with the risk of decreased serum albumin levels. Nonetheless, in our study, the decreased serum albumin levels were not independently associated with *H. pylori* infection in patients with pSS. The principal reason behind the contrary findings might be the small sample size in our study. In the occurrence and development of gastric cancer, previous reports indicated that *H. pylori* could significantly secret γ‐GT, in which the γ‐GT gene of *H. pylori* could remarkably promote gastric carcinogenesis by activating the Wnt signal pathway through upregulating TET1.^19^ It should be noted that the incidence of gastric cancer in *H. pylori*‐infected patients with pSS can be reduced. Moreover, in our study, it was observed that the levels of hypergammaglobulinemia, γ‐GT, hypertension, female sex and Anti‐SSA/Ro60 positive were independent factors of *H. pylori* infection in patients with pSS. To our knowledge, these findings are reported for the first time.

To exclude a low titer ANA positivity, the ANA positivity was initially defined in our cohort of pSS patients as a 1:320 titer or higher due to the lack of specificity in the old people or subjects with other chronic diseases. Further, it was suggested that the 1:320 of ANA positivity and RF (particularly IgA type of RF) were related to the presence of anti‐SSA antibodies^20^ In our study, the high titer ANA positivity was common in the *H. pylori*‐infected pSS patients. In this context, the ANA‐positive subgroup showed augmented levels of IgG, RF, and LSGB, consistent with the previous reports.^21^ In addition, it was observed that the levels of SSA, Ro52, SSB, creatinine, and uric acid were increased in the ANA‐positive subgroup.

Indeed, the highly proliferative polyps are the most commonly observed gastric polyps in patients in China. Previous reports indicated that 30 of 49 patients with proliferative polyps were recognized as *H. pylori*‐positive subgroup.^22^ Based on our statistical data, it was observed that only 19 cases (18.3%) of proliferative polyps were found in a total of 104 patients without *H. pylori* infection. However, there were only 3 cases accounting for 5.2% of proliferative polyps in 58 patients with *H. pylori‐positive* infection in all 162 patients. The reason could be due to the limited number of cases or gland atrophy caused by pSS. Considering these outcomes, we stress that further investigations are required to explore their reasons. Nevertheless, a consistent conclusion should be drawn that the primary pathological mechanism of *H. pylori* must be based on gastric mucosal apoptosis due to the overexpression of autoantibodies and gastric mucosal damage caused by self‐produced toxins. Moreover, previous reports demonstrated that the pSS patients infected with *H. pylori* resulted in higher rates of intestinal metaplasia and atrophy of gastric mucosa than *H. pylori‐*negative pSS patients.^23^, ^24^

In general, the salivary gland atrophy is confirmed by histological observations of LSGB. In this framework, the generation of significant lymphocytes in LSGB, with a FS value of ≥1, plays a dominant role in AECG and ACR classifications.^24^ Nonetheless, the sensitivity and specificity attributes of common salivary gland atrophy to pSS are not explicitly demonstrated. To a considerable extent, in this study, the relationship between salivary gland atrophy and *H. pylori*‐positive pSS patients has been detected, which, however, requires further investigations to understand the mechanistic views and pathogenesis better. In comparison among various histopathological subgroups, a phenomenon of positive histopathology, along with higher levels of IgG, ANA, SSA, Ro52, SSB, and uric acid, were observed in most of the *H. pylori*‐infected pSS patients, indicating a strong correlation between the pathological characteristics of the patients and the severity of the disease. Notably, these observations in this study were in agreement with the whole pSS cohort reported in previous studies.^25^, ^26^

Although it is challenging to predict the *H. pylori* infection in the body, it can be observed as it directly affects the prognosis of pSS patients. The univariate and multivariate analyses in this study indicated that the levels of hypergammaglobulinemia, were significantly correlated with the pSS in the *H. pylori* infection group. Notably, our study also confirmed that the levels of ALP were negatively correlated with pSS in *H. pylori* infection. Despite the establishment of correlation among these components, the specific reasons and mechanisms are still unclear, requiring further research. ALP, one of the vital hydrolases widely distributed in various organisms, is a critical biomarker often strongly associated with many physiological and pathological processes.^27^ Moreover, ALP is an essential functional enzyme in dental osteoblasts, playing a vital role in the pathogenesis of periodontitis.^28^ Previous reports indicated that the reduced ALP levels in invasive periodontitis cases could be attributed to impaired functions of neutrophils^29^ At the same time, it was reported that *H. pylori* infection was negatively correlated with allergic diseases (including atopic dermatitis, asthma, and allergic rhinitis). In this case, a significant negative correlation was established between ALP levels and eosinophilic esophagitis (EOE).^30^ These findings substantially guided in establishing a correlation that this enzyme played a protective role in xerosis patients with *H. pylori* infection, requiring further longitudinal studies to explore the detailed mechanistic views.

Despite the success in establishing the correlation between pSS and *H. pylori* infection, there exist some limitations in this study, requiring further studies to resolve them. First and foremost, it is highly challenging to obtain some critical information or variables, such as the ESSPRI score, as it is a retrospective study. Moreover, it is tough to confirm the causal relationship with a severe illness due to the limitation of the correlation analysis. Second, although the data of this study were collected in a tertiary hospital for 9 years, the sample size represented just one center and was mainly restricted to one location. Further investigations with multiple locations with improved sample sizes may result in a better conclusion. In addition, this study did not consider other traditional risk factors, such as age, hypertension, and hyperglycemia. We believe these factors may also play a vital role in the development of the disease. To address these limitations, multicenter prospective studies are further required to investigate the risk factors in the future.

## 5 CONCLUSION

In summary, this study has significantly explored the correlation between *H. pylori* infection and pSS concerning the clinical characteristics and risk factors in the patients registered for 9 years. Indeed, several infectious pathogens are often considered basic pathogenic stimuli to prompt and maintain immune system disorders, causing Sjogren's syndrome. Among them, various factors, including anti‐SSA/Ro60 positivity, hypergammaglobulinemia, reduced ALP, low γ‐GT, and hypercholesterolemia levels, were significantly associated with *H. pylori* infection in pSS patients. Contrarily, the levels of γ‐GT, hypergammaglobulinemia, hypertension, female sex and Anti‐SSA/Ro60 were identified as independent risk factors. In addition to establishing the relationship, these factors might provide some potential insights to improve clinical decision‐making, especially during the early stages of the disease.

## AUTHOR CONTRIBUTIONS

Ye He designed the study and wrote the first draft of the manuscript and conducted the statistical analysis. Lingzhen Hu and Wei Qiu performed the data collection and took part in statistical analysis. Lixia Zhu provided critical input into the data analysis and interpretation of the results. Xiaochun Zhu and Mingzhi Hong participated in conception, designed of the study and revised it critically for important intellectual content. All authors have read the draft critically to make contributions and also approved the final manuscript.

## CONFLICT OF INTEREST STATEMENT

The authors declare no conflict of interest.

## Supporting information

Supporting information.

## Data Availability

The data is available upon reasonable request.
