# Supplementary material for: Clinical characteristics and risk factors of Helicobacter pylori infection‐associated Sjogren's syndrome
Source: Immun Inflamm Dis. 2023 Oct 30;11(10):e994. doi: 10.1002/iid3.994 (PMC10614117; doi:10.1002/iid3.994)
Supplement: Supplementary file 1 — Supporting information. [file IID3-11-e994-s001.docx]

**Supplementary Table 1.***Analysis of multiple features of H. pylori-negative pSS according to ANA positivity.*

| **Characteristics** | **Total**  **(n= 130)** | **ANA-low**  **(n = 69)** | **ANA positive-high**  **(n = 61)** | ***P*-value** |
| --- | --- | --- | --- | --- |
| Female sex, n (%) | 111 (85.38) | 60 (86.956) | 51 (83.61) | 0.589 |
| Age, median years (mean ± S.D.) | 55.02±12.16 | 52.87± 1.408 | 57.44±1.57 | 0.32 |
| Hypertension, n (%) | 27 (20.77) | 15 (21.734) | 12 (19.67) | 0.77 |
| ESSDAI | 8.5 (3-19) | 7(3.0,19.5) | 9 (3.5,19.5) | 0.434 |
| Disease duration (months) (IQR) | 12 (5-36) | 12 (5-40) | 12 (6-24) | 0.143 |
| LSGB, lymphocytic focus ≥1, n (%) | 77 (59.23) | 41 (59.42) | 36 (59.02) | 0.963 |
| Anti-SSA/Ro60 positive, n (%) | 67 (57.69) | 34 (50.72) | 33 (65.57) | 0.583 |
| Anti-Ro52 positive, n (%) | 84 (64.15) | 40 (52.17) | 44 (78.69) | 0.092 |
| Anti-SSB positive (%) | 24 (18.46) | 9 (13.04) | 15 (24.59) | 0.09 |
| IgG levels, (g/L) (IQR) | 15.15 (13.07-18.90) | 14.90(12.45-17.75) | 15.500(13.65-21.00) | 0.207 |
| Hypergammaglobulinemia (> 16 g/L), n (%) | 57 (43.84) | 28 (40.58) | 29 (47.54) | 0.425 |
| IgA levels (g/L) (mean ± S.D.) | 2.98(2.23-3.817) | 2.99(2.20-3.805) | 2.97(2.325-3.915) | 0.922 |
| IgM levels (g/L) (IQR) | 1.25 (0.908-1.735) | 1.160 (0.870-1.580) | 1.25 (0.90-1.82) | 0.203 |
| C3 level (g/L) (IQR) | 1.07 (0.93-1.17) | 1.066 (0.935-1.180) | 1.02 (0.92-1.135) | 0.085 |
| C4 level (g/L) (IQR) | 0.23 (0.18-0.26) | 0.23 (0.19-0.265) | 0.22 (0.165-0.255) | 0.290 |
| RF positive, n (%)＞15,9IU/mL | 73 (56.15) | 28 (40.58) | 35 (57.38) | 0.056 |
| **WBC (× 10^9^/L) (IQR)** | 5.21(4.27-6.34) | 5.47 (4.61-6.83) | 4.51 (3.84-5.50) | **<0.01** |
| **Neutrophil numerals (× 10^9^/L) (IQR)** | 2.77 (2.14-4.07) | 3.10 (2.45-4.47) | 2.54 (1.87-3.80) | **0.018** |
| **Lymphocytes(× 10^9^/L) (IQR)** | 1.59 (1.17-1.92） | 1.64 (1.44-2.085) | 1.40 (0.95-1.79) | **<0.01** |
| Hemoglobin (g/L) (IQR) | 126.00 (117.75-134.25) | 127 (117.5-136) | 124(117.5-143) | 0.354 |
| **Platelet** (×10^9^/L) (mean ± S.D.) | 207.09 (169-245.45) | 223.20±74.71 | 187.47±64.50 | **0.04** |
| Albumin (g/L) (IQR) | 42.10 (36.83-44.40) | 42.20 (38.00-45.00) | 40.0 (35.6-44.0) | 0.204 |
| GPT (U/L) (IQR) | 18(13-28.73) | 16 (13-26) | 20 (14-38.5) | **0.030** |
| GOT (U/L) (IQR) | 23.50 (19-32.25) | 22 (19-28) | 26 (21-37) | 0.246 |
| γ-GT (U/L) (IQR) | (15-53.39) | 21(14-47) | 36 (19.5-60) | 0.053 |
| **ALP (U/L) (IQR)** | 77 (62-93.14) | 77 (59-92.20) | 76 (64-106.5) | **0.031** |
| SCr (μmol/L) (IQR) | 58 (51-65.25) | 57 (51-64) | 60 (51-60.5) | 0.583 |
| LDH (U/L) (IQR) | 217.39 (215.54-217.39) | 217.39(202-217.39) | 217.39 (206-217.39) | 0.090 |
| CRP (mg/l) (IQR) | 3.13(2.93-6.66) | 3.13 (2.77-5.94) | 3.14 (2.98-6.63) | 0.671 |
| ESR (mm/h) (IQR) | 21(9-27.25 | 18 (8-25) | 22.93 (10.50-32.00) | 0.33 |
| 1. dimer (mg/L) (IQR) | 1.45 (0.39-1.5) | 1.32 (0.34-1.45) | 1.45 (0.49-1.45) | 0.08 |
| Fasting blood glucose (mmol/L)(IQR) | 5.56 (5-5.80) | 5.5 (5.0-5.57) | 5.56 (4.95-6.0) | 0.379 |
| Uric acid (μmol/L) (IQR) | 273.50 (190.5-345.25) | 266 (223.5-357) | 386 (222-336) | 0.664 |
| Total cholesterol (mmol/L) (IQR) | 5.05(4.10-5.39) | 5.05 (4.21-5.45) | 4.84 (4.00-5.38) | 0.227 |
| Triglycerides (mmol/L) (IQR) | 1.43(0.98-1.59) | 1.40 (0.97-1.48) | 1.46 (1.00-1.48) | 0.841 |
| HDL(mmol/L) (IQR) | 1.23(0.97-1.31) | 1.23 (1.03-1.31) | 1.41 (0.82-1.41) | 0.331 |
| LDL(mmol/L) (IQR) | 1.23 (0.97-1.31) | 3.35 (2.31-4.01) | 3.23 (2.50-3.81) | 0.225 |
| creatine kinase (U/L) (IQR) | 88(45-108.65) | 105 (57-108.65) | 73 (43.5-108.65) | 0.726 |

Notes: Bold indicates P<0.05.

**Abbreviations**- ANA: antinuclear antibodies; C3: complement 3; C4: complement 4; CCR: creatinine clearance rate; CRP: C-reactive protein; ESR: erythrocyte sedimentation rate; ESSDAI: European League Against Rheumatism Sjögren’s syndrome disease activity index; LSGB: labial salivary gland biopsy; RF: rheumatoid factor; SCr: serum creatinine.

Supplementary **Table 2.***Analysis of multiple features of H. pylori-negative pSS according to histological pattern of LSGB.*

| **Characteristics** | **Total**  **(n = 130)** | **LSGB negative**  **(n = 53)** | **LSGB positive**  **(n = 77)** | ***P*-value** |
| --- | --- | --- | --- | --- |
| Female sex, n (%) | 111 (85.38) | 47 (88.69) | 64 (83.11) | 0.378 |
| Age, median years (mean ± S.D.) | 55.02±12.14 | 55.01±12.035 | 55.01±12.29 | 0.995 |
| Disease duration (months) (IQR) | 12.00 (5.00-36.00) | 12.00 (7.00-33.50) | 12.00 (5.00-36.00) | 0.890 |
| Fatigue, n (%) | 24 (11.54) | 11 (20.75) | 13 (16.88) | 0.576 |
| ESSDAI(IQR) | 8.5 (3.00-19.00) | 9.00 (4.50-19.00) | 7.00 (3.00-19.50) | 0.685 |
| ANA-positive, ANA ≥1:320,n(%) | 61 (46.92) | 25 (47.16) | 36 (46.75) | 0.936 |
| Anti-SSA/Ro60 positive, n (%) | 67 (57.69) | 30(56.60) | 37 (48.05) | 0.338 |
| Anti-Ro52 positive, n (%) | 84 (64.62) | 33 (62.26) | 51 (66.23) | 0.642 |
| Anti-SSB positive, n (%) | 24(18.46) | 11 (20.75) | 13 (16.88) | 0.139 |
| Hypergammaglobulinemia (> 16 g/L), n (%) | 57 (26.15) | 29 (54.72) | 28 (36.36) | 0.038 |
| IgA levels (g/L) | 2.98 (2.24-3.82) | 3.09 (2.42-3.98) | 2.960 (2.22-3.73) | 0.229 |
| IgM levels, g/L(IQR) | 1.25 (0.91-1.74) | 1.15 (0.88-1.69) | 1.36 (0.97-1.76) | 0.175 |
| C3 level (g/L) (IQR) | 1.07 (0.93-1.17) | 1.07 (0.98-1.19) | 1.02 (0.90-1.14) | 0.264 |
| C4 level ( g/L) (IQR) | 0.23 (0.18-0.26) | 0.23 (0.19-0.28) | 0.23 (0.18-0.24) | 0.287 |
| RF(IU/mL) (IQR) | 13.45 (9.69-60.92) | 20 (11.01-60.92) | 11.50 (8.88-60.92) | 0.447 |
| WBC(× 109/L) (IQR) | 5.21(4.27-6.34) | 4.87 (3.64-6.53) | 5.25 (4.42-6.26) | 0.547 |
| Hemoglobin(g/L) (IQR) | 126.00 (117.75-134.25) | 126 (116.5-134) | 126 (118-135) | 0.354 |
| Platelet (×109/L) (IQR) | 207.09 (169.00-246.25) | 207.10 (155.50-253) | 207.10 (172.50-240.50) | 0.669 |
| GPT (U/L) (IQR) | 18.00 (13.00-28.73) | 16.00 (12.00-25.00) | 20.00 (14.00-28.73) | 0.230 |
| GOT (U/L) (IQR) | 23.50 (19.00-32.25) | 24.00 (19.00-29.00 | 23.00(19.00-34.58） | 0.906 |
| γ-GT (U/L) (IQR) | 25.50 (15.00-53.59) | 24.00 (14.500-51.00) | 27.00(16.00-55.79) | 0.070 |
| ALP (U/L) (IQR) | 77.00 (66.00-93.14) | 77.00 (60.00-96.00) | 77.00(62.50-92.19) | 0.327 |
| SCr (μmol/L) (IQR) (IQR) | 58.00 (51.00-65.25) | 57.00 (51.00-62.00) | 59.00(51.00-67.00) | 0.518 |
| Uric acid (μmol/L)(IQR) | 217.40 (190.50-217.39) | 261.00 (225.00-340.50) | 301.00(220.00-345.50) | 0.554 |
| CRP (mg/l) (IQR) | 3.13 (2.93-6.66) | 3.13 (2.98-6.66) | 3.13 (2.77-5.94) | 0.062 |
| ESR (mm/h) (IQR) | 21.00 (9.00-27.25) | 22.00 (10.50-33.00) | 18.00 (9.00-26.00) | 0.349 |
| D-dimer (mg/L) (IQR) | 1.45 (0.39-1.45) | 0.91 (0.31-1.45) | 1.45(0.51-1.45) | 0.225 |
| Fasting blood glucose (mmol/L) (IQR) | 5.56 (5.00-5.80) | 5.45 (4.85-6.15) | 5.56 (5.10-5.60) | 0.971 |

Notes: Bold indicates P<0.05.

**Abbreviations**- IQR: interquartile range; ANA: antinuclear antibodies; C3: complement 3; C4: complement 4; CRP: C‐reactive protein; ESR: erythrocyte sedimentation rate; ESSDAI: European League Against Rheumatism Sjögren’s syndrome disease activity index; ESSPRI: European League Against Rheumatism Sjögren’s syndrome patient reported index; LSGB: labial salivary gland biopsy; RF: rheumatoid factor; SCr: serum creatinine
